# Everyday (in)equality at home: complex constructions of gender in South African families

**DOI:** 10.3402/gha.v9.31122

**Published:** 2016-06-09

**Authors:** Rebecca Helman, Kopano Ratele

**Affiliations:** 1 Institute for Social and Health Sciences, University of South Africa, Pretoria, South Africa; 2 Violence, Injury and Peace Research Unit, South African Medical Research Council-University of South Africa, Tygerberg, South Africa

**Keywords:** violence, HIV, gender, families, South Africa

## Abstract

**Background:**

High rates of violence and HIV have been documented within the South African context. Constructions of masculinity and femininity that position men as dominant and highly sexually active and women as subordinate and acquiescent have been found to contribute towards gender inequality. This inequality is in turn related to negative health consequences, specifically violence against women, children, and other men, as well as sexual risk. Within this context it becomes important to explore how problematic constructions of gender are being (re)produced and how these constructions are being challenged. Families have been identified as key sites in which gender is both constructed and enacted on a daily basis and it is within this space that children are first exposed to notions of gender.

**Objective:**

This article draws from a study that was intended to expand on the limited understandings of the ways in which gender (in)equality is constructed and conveyed within the context of South African families on an everyday basis.

**Design:**

Children and parents in 18 families from a range of different material and cultural backgrounds were interviewed about the meanings and practices of gender within their homes. Data were analysed using a Foucauldian discourse analysis.

**Results:**

The data reveal how problematic constructions of masculinity and femininity are (re)produced but also challenged within a range of different families. Gender and gender (in)equality are therefore routinely accomplished in complex ways.

**Conclusions:**

These findings have important implications for promoting gender equality and therefore for disrupting violence and sexual risk as gendered health issues.

## Introduction

Within the South African context, where extremely high rates of violence and HIV have been documented, it is necessary to investigate the factors that shape these social and health epidemics. Violence and HIV are major public health issues disproportionately affecting South Africa (1, 2). Gender inequality, perpetuated through dominant and highly sexually active constructions of masculinity and subordinate and acquiescent constructions of femininity, which we refer to as *problematic constructions of gender*, has been identified as a significant factor in health and shapes practices of violence as well as sexual risk (3–5). A key site in which notions of masculinity, femininity, and gender equality are constructed and enacted is the family. Yet there is a surprising lack of research that has explored the ways in which gender and its potential health consequences emerge within the context of families in South Africa. In light of this lack, it is important to explore how problematic constructions of gender and the gender inequitable relations they enable are being (re)produced,^1^ as well as how these constructions may be being challenged within South African families.

It is important to recognise the particular ways in which gender inequality and violence are represented in both public and academic discourse within the South African context. Gender inequality (particularly in relation to male violence and sexual risk) has tended to be represented both in the media and much academic literature as a ‘poor black problem’. For example, Anene Booysen^2^ was referred to as ‘a poor black girl who had been raped’ (8).

Several studies have focused on the ways in which violence and sexual risk are constructed as normative among poor black men and women in various parts of the country (9–11). We do not deny that various social, cultural, and economic factors such as racialised poverty and neighbourhood structures shape practices of violence and sexual risk in very particular and unequal ways within the South African context. However, representations of these practices as only affecting certain groups not only pathologises these groups in harmful ways, but also serves to disguise the ways in which gender inequality is a profoundly powerful factor at all levels of our society. The examples of the model Reva Steenkamp, killed by her Paralympian boyfriend Oscar Pistorius, and Jade Panayiotou,^3^ whose husband is awaiting trial for allegedly hiring contract killers to carry out her murder, demonstrate that violent practices of gender inequality are not limited to the poor, black, and working class (even though these cases may be represented differently in public discourse^4^). This is supported by research evidence that suggests that rates of murder by intimate partners are high among all groups of South African women (12). The South African context should therefore be viewed as ‘a patchwork quilt of patriarchies’, in which inequitable gender relations extend beyond the social categories of race, class, and culture (13, p. 155).

### Gender, violence, and HIV/AIDS within the South Africa context

It has been well documented that extremely high levels of violence and HIV exist in South Africa. In a study conducted across three provinces, it was found that 24.6% of women had experienced domestic violence (14). Police statistics reveal that between 1 April 2014 and 31 March 2015, 62,649 sexual offenses were reported nationwide (15). Estimates indicate that 12.2% of the population (6,422,179 people) are living with HIV (16).

In light of these concerning rates, research has sought to explore the specific factors that shape these epidemics. Much of this research has focused on how gender, that is, particular constructions of masculinity and femininity, and gender power inequality fundamentally shape practices of violence (particularly intimate partner violence and rape), as well as the feminisation of HIV (17). Gender inequality, underpinned by problematic constructions of masculinity and femininity, contributes to practices of violence and sexual risk (18). Dominant constructions of masculinity that centre on men's power, control over others, and sexual entitlement scaffold men's use of violence against women and children, as well as other men (7). Men's use of violence represents an attempt to ‘secure a position of status which is central to the man's experience of being a man, and in this way is tied to societal expectations of manly behaviour’ (19, p. 18).

Gender constructions and inequality have also been related to the high rate of HIV infection. Gender constructions that prescribe female sexual passivity and ignorance as well as overpowering male sexual desire, at times said to be provoked by females, make it difficult for women to negotiate safe sex, while simultaneously placing men at risk of HIV (20). Moreover, men's gender power over women is associated with women's chances of contracting HIV (9). Therefore, these particular constructions can be seen to not only enable noxious relations between men and women, but also to create a context in which these unequal relations come to be understood as ‘normal’ and ‘natural’. Collectively, these findings point to the promotion of gender equality as an important health issue, particularly in disrupting practices of violence and sexual risk. Therefore, there is a need to closely explore the ways in which problematic constructions of gender are being (re)produced, as well as how they are being challenged within South African family contexts.

### (Re)productions of gender inequality

A particularly concerning finding of South African research is that children are involved in constructing gender in problematic, unequal ways from a young age. Literature indicates that children are aware of gender from the age of two (21). Numerous studies in South Africa have investigated the ways in which children construct gender in unequal ways, as well as how they police the boundaries between the genders (3, 22). Research has also found that inequitable and opposing constructions of masculinity and femininity fundamentally shape young people's notions and experiences of sexuality (4). This research highlights the fact that children and young people construct gender in these problematic ways, thereby (re)producing gender inequitable relations. Therefore, there is a need to develop clearer understandings of how and why gender inequality is (re)produced within the South African context. Simultaneously, it is necessary to investigate how it may be possible to reconfigure these constructions in more egalitarian ways.

Families exist as key sites in which notions of gender are constructed and enacted (23). Many feminist scholars have argued that families operate as gender mills, through which hierarchical gender relations are reproduced (24). Within the context of the family, various discourses can be seen to position men and women in unequal ways. For example, discourses of ‘natural mothering’ (25), which position women as possessing innate nurturing capacities, can be seen to make women primarily responsible for the care of children. Conversely, men are exempt from caring activities by virtue of their positions as providers, protectors, and disciplinarians (26). Research has documented the ways in which these discourses produce unequal relations within families (27, 28). However, there is also evidence to suggest that more equal constructions of gender are beginning to emerge (29, 30).

Studies have also documented the ways in which parents send very clear messages to their children about gender, for example through purchasing particular toys and clothes (31) and discouraging or encouraging children's participation in particular activities (32). Family studies have also shown how children's views about gender are correlated with those of their parents (33, 34). This evidence highlights the centrality and power of gender within the context of families and suggests that they are important spaces in which to explore practices of (in)equality. However, the majority of the research that has explored gender within the context of families has been conducted in the United States.

Despite substantial evidence suggesting that families exist as inherently gendered spaces, as well as considerable evidence linking particular constructions of gender to concerning epidemics of violence and HIV/AIDS, there is a surprising lack of research that has explored the nuanced ways in which gender is constructed within South African families. This qualitative, critical gender study is thus intended to expand on the limited understandings of how gender is constructed within a range of different families in South Africa, as well as to explore how these constructions are related to practices of gender (in)equality. This study appears particularly important within the South African context, where, despite constitutional gender equality, inequality between men and women (particularly in the form of high rates of violence and HIV) continues to fundamentally shape the daily lives of millions of children and adults. It is within this context that it becomes imperative to more closely investigate the ways in which both children and adults are (re)producing gender inequality, as well as how they may be attempting to reconfigure gender relations in order to promote equality.

## Objective

Based on the gaps we identified in the literature, this study had two primary aims. First, we examine how gender is constructed within South African families by both parents and children. Second, we are interested in how these particular constructions are related to practices of gender equality and inequality.

## Method

### Participants

This article is based on a qualitative, critical gender study that received ethical clearance from the University of South Africa. Eighteen South African families were interviewed about the meanings and practices of gender within their homes. All the families included in the study had children between the ages of 6 and 17 living in the house. Children younger than six were considered too young to able to answer the interview questions in detail. However, in cases where younger children were present in the house, they were allowed to participate in the interviews. We recruited families in a number of different ways in order to ensure the inclusion of a variety of different family structures and racial groups. This recruitment strategy was also intended to ensure the inclusion of families who construct gender in different ways. In order to recruit families who were likely to identify with notions of gender equality, we used a purposive sampling technique and posted an advert on two social media pages that focus on feminist issues. Families were also purposively recruited through religious and community-based organisations. The first author asked two former colleagues, one who is a member of a church congregation in a middle-class suburb in Cape Town and the other who is a member of a community-based organisation in a working-class suburb, to recruit families. A snowball sampling technique was also employed, with families who agreed to be interviewed being asked to refer other families that they knew. All the families who responded to the online advert and who were contacted through the religious and community-based organisations agreed to participate in the study.

### Procedure

Families who contacted the first author, expressing an interest in being interviewed, were provided with further details of the study, with some parents requesting a copy of the interview schedule prior to being interviewed. Interviews were then arranged at a time and location that was convenient for the family. All interviews ended up being conducted at families’ homes. Parents were required to sign consent forms for both themselves and their children, whereas children completed assent forms. The interviews lasted between 10 min and 1.5 h (with parents’ and older children's interviews tending to last longer than those with younger children^5^). Parents and children were given the option of being interviewed separately or together. Following the interviews, participants were given the option to view the interview transcript or listen to the interview recording.

All the interviews were conducted in English. The majority of the interviews were conducted by the first author, a white woman. However, just over a third of the interviews were co-conducted by the second author, a black man. All interviews were transcribed by the first author. The interview questions covered a range of topics related to meanings and practices of gender within the family. The interviews were semi-structured, with some questions asking about general views relating to gender and gender equality (for example, parents were asked, ‘Do you think there should be a difference in how boys and girls are raised?’ and children were asked, ‘Do you think boys and girls should be treated differently?’). Other questions asked about specific family practices (for example, parents were asked, ‘How do you think the fact that your child is a boy/girl affects the way you raise him/her?’ and children were asked, ‘Do you think the fact that you are a boy/girl affects the way in which your parents treat you?’).

### Data analysis

Consistent with our critical interest in the ways in which gender is constructed and how these constructions promote or challenge gender inequitable relations, a Foucauldian discourse analysis (FDA) was used to analyse the interview transcripts. FDA aims to enable an explanation of ‘the working of power on behalf of specific interests and [an analysis of] the opportunities for resistance to it’ (35, p. 41). This is done through examining the ways in which the social world is constituted in particular ways through discourse and how language serves to not only produce particular meanings but also particular objects and subjects (36, 37). Unlike discursive psychology, which is concerned with how discursive resources are used in relation to issues of interest and stake (38), issues of power and authority are central in FDA. Therefore, attention is paid to dissecting, disrupting, and rendering the taken-for-granted perceptions and assumptions strange by interrogating ‘the discourses of true and false … the correlative formation of domains and objects … the verifiable, falsifiable discourses that bear on them, and … the effects in the real to which they are linked’ (39, p. 237).

Our analytic approach was also shaped by an awareness that social categories of class, race, age, and others, intersecting with and co-constitutive of gender, profoundly shape people's health. For example, in South Africa young black females aged 20–34 years are at elevated risk of HIV (2). The imbrication of and dynamics between different social categories make approaches informed by the concept of intersectionality (40) of great value in attempting to understand and represent the relationship of gender constructions, gender equality, and health within this particular context. Intersectional approaches pay attention to the ways in which these categories co-produce each other and, rather than being additive, have overlapping effects on health. The concept of intersectionality provides health researchers a richer understanding of health and other inequalities as well as better contextualised intervention strategies, thus ascertaining results that are more easily accepted and effective within communities (41). Some health researchers indicate that using an intersectional gender-power analysis of health issues is more effective than approaching phenomena such as violence against women and girls as a single-cause issue that looks the same everywhere (42). In our analysis, we explore the ways in which problematic constructions of gender were (re)produced as well as challenged within a range of different families, and how gender intersects with other social categories in producing particular discourses on gender (in)equality.

The process of analysis involved identifying discursive constructions of gender, locating these various discursive constructions within broader discourses, and closely examining the discursive contexts within which the different constructions were deployed. Following this, the subject positions offered by the discursive constructions were identified. The ways in which particular discursive constructions and the subject positions they enable make certain actions possible, while rendering others impossible, were then explored. Finally, the relationship between discourse and subject positions was examined (43). In repeated discussions between the authors, which took place immediately following each interview, as well as after multiple, separate readings of the interview transcripts, the discourses were grouped into broad themes.

## Results

In this section, we begin by presenting a descriptive analysis of the 18 families studied; we then present the themes drawn from the interviews.

### From more patriarchal to less patriarchal families

Table 1 provides basic demographic information for each family. Initially families were asked to classify themselves as either ‘egalitarian/feminist’ or ‘patriarchal/traditional’. However, as we began to analyse the data it became clear that these two categories did not meaningfully capture the ways in which gender was being constructed in these 18 families. Rather than some families operating as ‘egalitarian/feminist’ and others as ‘patriarchal/traditional’, the data suggest that these categories are fluid rather than fixed. We suggest a more accurate way of categorising families is on a spectrum, from more patriarchal to less patriarchal. In other words, there are no wholly egalitarian or feminist families and no absolutely patriarchal or traditional families. As we attempt to demonstrate below, all families in differing ways (re)produced and challenged problematic constructions of gender simultaneously. Therefore, instead of separating and defining families in limiting ways, we focus on the common ways in which families constructed gender as it intersects with other social categories of analysis.

**Table 1:** Family characteristics

Family composition	Family members interviewed	Race^a^	Class	Self-defined categorisation
Single mother, two daughters (21 and 25), son (16) (children see father occasionally)	Mother, son	White^b^	Middle class	Feminist^c^
Two-parent nuclear family, two daughters (10 and 13)	Mother, both daughters	White	Middle class	Feminist
Mother, father, two daughters (8 and 9) (youngest daughter is adopted) living with another married couple (polyamorous)	Mother, father, both daughters	White; adopted daughter is black	Middle class	Feminist
Single mother and son (7) (shared custody with ex-husband)	Mother, son	White	Middle class	Feminist
Single mother and son (13) living with mother's female partner (child sees father on weekends)	Mother, son	White; mother's partner is black	Middle class	Feminist
Two-parent nuclear family, three daughters (4, 8, and 9)	Mother, father, all three daughters	Mother is white, father is black, children are mixed race	Middle class	Traditional and God-focused^d^
Two-parent nuclear family, son (8), and daughter (6)	Mother, father, both children	White	Middle class	Traditional
Two-parent nuclear family, son (4), and daughter (8)	Mother, father, both children	Father is white, mother is coloured, children are mixed race	Middle class	Equal^e^
Single mother and son (8) (no involvement from father)	Mother, son	Coloured	Working class	Traditional
Single mother, daughter (7) (no involvement from father)	Mother, daughter	Mother is coloured; daughter is mixed race	Working class	Feminist
Grandmother, mother, three sons (4, 10, and 14) (boys have limited contact with fathers)	Grandmother, mother, two sons (10 and 14)	Coloured	Working class	Equal
Two-parent nuclear family, four daughters (15, 19, 21, and 23)	Mother, daughter (15)	Coloured	Working class	Traditional
Grandmother, aunt, two uncles, granddaughter (8) and grandson (7)	Grandmother, both children	Coloured	Working class	Traditional
Two-parent nuclear family, four sons (11, 15, 17, and 20); niece (16) spends afternoons and weekends	Mother, son (11), and niece (16)	Coloured	Working class	Traditional
Grandmother, grandfather, aunt, granddaughter (19), and grandson (16)	Grandmother, grandson	Coloured	Working class	Traditional and religious
Mother and father, four daughters (4, 10, 13, and 19), aunt, niece, grandmother	Mother, three daughters (10, 13, and 19)	Black	Working class	Religious
Two-parent nuclear family, two daughters (1 and 16)	Mother, father, daughter (16)	Black	Working class	Traditional and religious
Single mother and son (11)	Mother, son	Black Namibian	Middle class	Traditional

a Priority has been given to race in the table (with families being grouped according to race). This was done in order to demonstrate the diversity of the families interviewed. However, families could also have been grouped according to class or family structure.

b The terms ‘coloured’, ‘black’, and ‘white’ were socially constructed racial categories used under the system of apartheid in South Africa to classify people according to their race. The issue of how to represent our participants has been one fraught with conflict, contradiction, and confusion. On the one hand we have found it difficult to move beyond the apartheid racial categories. To a large extent our classification of families on both race and class were done subjectively. However, this subjective classification took into account a range of different factors (including how participants described themselves and their families, observations of their living arrangements, and discussions of their occupations). It is important to note that as a result of the legacy of apartheid, including laws such as the Group Areas Act which demarcated suburbs based on race, class, and race, continue to be closely tied together. This is reflected, for example, in almost all the ‘coloured’ families also being ‘working class’. While we acknowledge that this method of classification is problematic in that it reifies these harmful, divisive categories, we feel that this classification remains necessary. We also do not believe that our subjective classifications are arbitrary. For example, the latest statistical survey of the community from which a group of our participants were drawn supports our classification of them as ‘coloured’ and ‘working class’. This study showed that 94% of the community is ‘coloured’ and 79% have a monthly household income of less than R 6,400 (US$425) (44).

c See ‘Participants’ section for a definition of *feminist* and *traditional* families.

d Within the context of the study, families used the terms *God-focused* or *religious* to refer to their families as being guided by biblical principles. In some instances, this included a patriarchal division of labour (father as head of household, mother as caregiver).

e Some families problematised the term *feminist* and instead defined themselves as *equal*.

An analysis of the interview transcripts revealed three broad themes: (re)production of problematic constructions of masculinity and femininity; disruptions of problematic constructions of masculinity and femininity; and non-binary constructions of gender. Due to space constraints, for the purpose of this paper we focus primarily on the first two themes. However, we do touch on non-binary constructions of gender as they relate to disruptions of problematic constructions of masculinity and femininity.

Through the analysis we attempt to demonstrate the ways in which problematic constructions of gender were both (re)produced and challenged in a range of different families. We also pay attention to the ways in which participants’ intersecting identities shaped their constructions of gender in particular ways.

### (Re)productions of problematic constructions of masculinity and femininity

In all 18 families, there were instances in which both children and parents constructed men and women as essentially different from one another.

Caleb^6^: Coz they … they the girl. They must know better. They must do more chores than boys.

In the above extract, Caleb, a 14-year-old coloured boy who lives with his grandmother, mother, and two brothers in a working-class community, constructs girls and boys as inherently different. Caleb makes a direct connection between female sex (‘they the girl’) and female nature (‘they must know better’). Here he can be seen to be drawing on an essentialised biological discourse that positions ‘femaleness’ as fixed and inextricably linked to the female body (45, 46). The use of the word ‘must’ serves to construct this female nature as binding and immovable. This female nature is also directly connected to a specific role within the context of the household (‘they must do more chores than boys’). Therefore, this discourse serves to fix men and women in unequal positions within the family, with women being made responsible for domestic chores. Research has documented the ways in which domestic chores, caring for children, and providing emotional support continue to be regarded as ‘women's work’ (28). The continued power of these social understandings can be seen to discourage men from engaging in these types of activities and therefore from disrupting practices of inequality within their homes (47).

Caleb's reference to ‘chores’ suggests that his construction of girls is produced by his intersecting identities of gender, age, and class. The practice of children carrying out household chores occurs primarily in poor and working-class households and usually falls on female children in particular (48). Therefore, Caleb's discourse, which positions girls as responsible for chores, appears to be shaped by his position as a child living in a low-income community.

As can be seen from Caleb's narrative above, constructions that position men and women as essentially different from each other have important implications for practices of power and (in)equality within the home. In many interviews, both parents and children positioned men and women as needing to occupy clear (and different) roles in the family.

Interviewer: And who's the boss in your family?

Elijah: Me

Interviewer: You? [Charmaine laughs] … and why do you say you're the boss?

Charmaine: What do er mommy always tell you about … why you the boss?

Elijah: Because I'm the man in the house.

When asked who the boss is in his family, Elijah, a coloured boy of eight who lives with his mother Charmaine, prompted by her, notes that he is. When asked to clarify this, Elijah notes that it is because he is ‘the man in the house’, thereby making a direct connection between maleness and authority. Here Elijah can be seen to be drawing on a discourse of male authority that positions men as ‘naturally’ occupying a position of power within the context of the family (47, 49). Much South African research has documented how, in a variety of different contexts, men continue to be regarded as the head of the household (50–52). This discourse of ‘unquestioned’ male authority can be seen to have important implications, not only within the context of South African families, but also within South African society more broadly. Studies in South Africa as well as in other parts of the world have demonstrated the ways in which constructions of male authority are related to practices of violence both within and outside the home. For example, it was found that the discourse of male authority served to rationalise the use of violence by fathers against their children in communities in Cape Town (50).

Elijah and Charmaine's discussion, through which a discourse of male authority is produced, appears to be shaped by the intersection of their racial and class identities, as well as their family structure. The particular community in which Elijah and Charmaine live is characterised by high rates of father absence, with other research highlighting how this is regarded as particularly problematic for male children in this community, who are seen as lacking an authoritative figure to guide and discipline them (53). Therefore, broader understandings of fatherhood and masculinity that are prevalent in this coloured, working-class community, coupled with the absence of Elijah's father, have co-produced a discourse that positions him as the male authority in his household.

As the extracts above illustrate, problematic constructions of masculinity and femininity were prevalent in participants’ narratives. These constructions operated to position men and women as essentially different from one another, as well as to fix them in unequal positions within the family. However, present in participants’ narratives were also instances of ambiguity and conflict, in which these problematic constructions were partially disrupted.

Aaron: Well definitely, I mean … ca-can't exactly treat them the same if they, um, do if they, you know, testosterone versus like [laughs] you know? You've gotta handle them in different ways. I mean I myself am coz […] I've been raised by women […] I tend to be more, um, calm and respectful.

Although Aaron, a 16-year-old white boy living with his mother in a middle-class suburb, constructs men and women as essentially different by virtue of their biology (‘testosterone’), he also constructs his ‘masculine nature’ as fluid rather than fixed. He notes that he is ‘calm and respectful’. Here he can be seen to be constructing his masculinity in opposition to ‘traditional’ ideals of power, strength, dominance, and aggression that have been strongly connected to male biology (54). He also attributes his calmness and respect to the fact that he has been ‘raised by women’. Therefore rather than constructing masculinity as biologically fixed and immoveable, he constructs it as socially constituted and fluid. In this way, the connection between male biology and male nature is to some extent disrupted. Through this, space is created for alternative, less problematic masculinities to emerge and the notion of ‘natural’ maleness is unsettled.

The biological discourse that Aaron draws on, through his use of the word ‘testosterone’, can be seen to be shaped by an intersection of his age and education. In comparison to the younger children from different racial and class backgrounds discussed above, Aaron draws on a scientific notion of male hormones. This reflects both his mental development as a teenager and his exposure to privileged, private school education.

### Disruptions of problematic constructions of masculinity and femininity

As demonstrated above, in some families problematic constructions of masculinity and femininity seemed to predominate, with some instances in which both parents and children attempted to disrupt these rigid constructions. However, in a number of other families both parents and children openly challenged the notion that boys and girls should be treated differently or that inherent differences exist between the two sexes. These disruptions of problematic constructions of masculinity and femininity were more prevalent and extensive among familiesthat identified as feminist or ‘gender equal’. However, as discussed below, they also occurred in traditional and religious families.

Interviewer: Do you think parents should treat boys and girls differently?

Nomhle: They can treat them the same.

Interviewer: And why do you think they can treat them the same?

Nomhle: Because boys and girls can do the same thing […] Boys can play with Barbie, girls can play with Batman.

In contending that ‘boys and girls can do the same thing’, Nomhle, an adopted black girl of 8 from a middle-class family, is offering a basic argument for gender non-discrimination. This contention is further reinforced by Nomhle positioning Batman (which embodies various aspects of traditional masculinity including physical prowess and technological ingenuity) as an appropriate girls’ toy and Barbie [which embodies various aspects of emphasised femininity, including accommodating the desires of men (55)] as an appropriate boys’ toy. Therefore Nomhle refutes the binary classifications of gender and begins the work of reformulating gendered discourses by ‘combining two previously dichotomized discourses of gender’ in order to make new (and more equal) subjectivities available to both boys and girls (56, p. 114). Therefore, she can be seen to be making space for more flexible and egalitarian notions of gender.

Nomhle's reference to the toys Batman and Barbie can be seen to be co-produced by an intersection of her age and class identities. The centrality of these objects within Nomhle's discourse is likely due to both her developmental phase, namely, the fact that she is a child, and to her family's socio-economic position, so she might well have access to these kinds of toys. It is interesting to note that a number of other middle-class children of a similar age used the examples of toys to construct gender in particular ways.

Nomhle's father Daniel also attempts to disrupt essentialist discourses of gender in his discussion of how he parents his two daughters.

Daniel: I mean, I think if you, if you're basing how you act towards your children based on gender, perhaps you're not listening closely enough to their individual voices. I mean, I have two girls but I certainly don't bring them up in the same way because they have vastly different needs and vastly different personalities.

Daniel discursively constructs parents who treat their children in a particular way by virtue of their gender as ‘not listening closely enough’ to the children's individual voices. Here Daniel constructs parents who treat their children in a particular way by virtue of their gender as careless and even harmful. Linked to this, through noting that despite the fact that he has two daughters, ‘they have vastly different needs and vastly different personalities’, Daniel disrupts the biological discourse of femininity, which constructs all women as essentially the same due to their shared physiology.

Daniel's attempt to disrupt binary, essentialist notions of gender appears to be a result of the intersection of his class, culture, and gender identities. The use of the expression ‘listening closely’ to children suggests a child-centred approach to parenting, which is primarily Western and middle class. Coupled with this, Daniel's use of a discourse that challenges essentialist notions of gender also appears to be linked to his identity as a queer man. In his discussion of his position as a man, he similarly refutes the binary categories of ‘male’ and ‘female’ and notes ‘I don't fit into male gender roles at all’. Therefore, Daniel's positions as a white, middle-class, genderqueer man co-produce his gender-equitable discourse.

Similarly to Nomhle and Daniel, Pamela, a black mother of four living with her husband, her mother, her sister, and her sisters’ two children in a township, disrupts the rigid binary between male and female work.

Pamela: If it's a boy he can do everything, he doesn't have to cry and say, oh I don't have nobody to help me. Coz he's used to the girls, used to cook and wash dishes so now he's g– he must go to work only. No! Like my husband he can do everything. He can clean the house, he can cook, he can make bread.

Here she constructs men as being able to do housework, which in a range of different cultures has historically been regarded as women's work (28, 57), as practical and necessary. Although she does make reference to the fact that men are ‘used to’ women cooking and washing the dishes, implying that this order of things is normative, she challenges the ‘naturalness’ of this construction through her definitive use of the word ‘No!’. This notion is further disrupted by her discussion of her husband, who she notes is able to participate in a range of domestic tasks within the household. Linked to this, she also refutes the notion that men should be primarily responsible for paid work (‘he must go to work only’). During the interview, when asked about who within the household was working, she noted that she, her husband, her sister, and her mother all worked and pooled their resources. They were also all responsible for helping with housework. Therefore, Pamela appears to be disrupting the unequal division of labour between men and women within the family, instead arguing for a more egalitarian situation in which both paid and household work are shared.

While, like Nomhle and Daniel, Pamela too is attempting to disrupt binary, unequal constructions of gender, it appears that her production of this discourse is shaped by different factors. Central to Pamela's discussion of gender equality is the issue of survival. She appears to be arguing that if boys and men do not engage in ‘girls’ work’, such as cooking, they will not be able to look after themselves. This particular construction of gender equality as necessary for survival can be seen to be linked to the intersection of Pamela's race, class, and gender positions. As a black, working-class woman she is among the most impoverished group in South Africa (58). Therefore, it is not unanticipated that the notion of practical survival emerges as central within her discourse of gender and gender equality.

Through challenging the rigid binary between masculinity and femininity and challenging unequal gender relations, a number of families disrupted problematic constructions of masculinity and femininity and made space for more egalitarian gender relations to emerge. However, within these same families there were also instances where masculinity and femininity were constructed in more rigid and unequal ways.

Patricia: The gender division of labour comes home to roost … *completely* when there are babies … in the house. Um … coz the unfortunate reality is that the buck does stop with mom […] I know in the first two years only mom can lactate basically so there is a … there's that […] I actually don't mind it too much […] it's OK that there's one parent who's more … kind of more cuddly […] and one parent who's their dad who winds them up at night just before bed time [laughs] and … and does fun things with them.

In this extract Patricia, a white mother of two living with her husband and children in a middle-class suburb, positions the mother as the primary caregiver. A direct connection is made between caregiving and female biology, with women's ability to lactate being constructed as a central component of women's ability to care for children. Associated with this, Patricia constructs mothers as more cuddly, drawing on a discourse of traditional femininity that positions women as more emotional and emotionally available to children (25). In contrast, the father is constructed as ‘the fun parent’, who is responsible for secondary rather than primary caretaking activities. This unequal family arrangement is discursively positioned as natural through Patricia referring to it as ‘the unfortunate reality’. This suggests that, although Patricia may be aware of the unequal nature of this arrangement, she regards it as natural and therefore unchangeable. Furthermore, she notes that she actually doesn't ‘mind it too much’, thereby constructing this as an acceptable dynamic. Therefore, although some families are disrupting problematic notions of masculinity and femininity, within these families inflexible and unequal constructions of masculinity and femininity continue to exist and shape relationships in unequal ways.

Patricia's narrative provides a particularly interesting example, not only of how problematic constructions of gender can be simultaneously challenged and (re)produced but also of how the intersections of various identities also produce particular constructions of gender in complex ways. In her interview, there were many instances in which Patricia challenged rather than (re)produced problematic constructions of gender. For example, she noted ‘I am not a very girly girl and my children are not very girly girls, [so] I mean I think kids should be parented according to where they are… there are some boys who want to run around […] and some boys that won't and some girl who want to run around’. Linked to this, in the above extract she makes reference to the ‘gender division of labour’, which is a feminist concept drawn from a Marxist perspective. Therefore, it appears that her gender equality discourses are produced through an intersection of her gender identity (‘not a very girly girl’) and her exposure to a certain level of feminist education. However, she also draws on a biological discourse that positions women as primarily responsible for childcare. This discourse appears to be linked to her intersecting identities as an educated subject and a mother. The use of the word ‘lactate’ calls to mind a scientific approach to mothering, which reflects a certain level of education (perhaps the kind that is gained by reading literature on motherhood).

## Limitations

We believe this study contributes to understanding the ways in which gender and gender (in)equality are constructed within the context of South African families. However, we also acknowledge that the study has a number of limitations. First, although through our sampling strategy we were able to recruit families from a range of different racial, class, and cultural backgrounds, as well as a diverse range of family structures, this strategy also resulted in a number of limitations. Because we accessed ‘gender egalitarian’ families via particular social media platforms, we were only able to access a subset of these types of families, those who belong to these social media groups. The families that responded to the online advert were predominantly, although not exclusively, white and middle class. Similarly, the community-based organisation through which families were recruited is situated within a community that is almost entirely coloured and working class. The lack of a wider range of families has limited our understanding of how certain intersecting identities shape constructions of gender in particular ways.

Second, although not all our participants spoke English as their first language, due to limited resources, all of the interviews were conducted in English. This can be seen as an important limitation, particularly in light of our method of analysis, as language is a central component within discourse. It is therefore important that future studies conduct interviews in participants’ home languages. However, we would also like to note that this does not entirely resolve the issue of language, as issues of translation (for example using concepts developed in one language in another language or translating interviews for transcription and analysis) also impact on language in important ways.

As should be apparent throughout the paper, the study has been fundamentally shaped by our positions as gender-critical researchers focused on the promotion of gender-equitable relations. The research questions, sampling of participants, interviews, and analysis have all been determined in relation to this orientation. We do not regard this as a limitation, as the research process is unavoidably shaped by the researcher (43). However, we wish to acknowledge that our particular orientation and interest in gender equality have been central to the study.

## Conclusions

This study set out to explore the ways in which gender is constructed in a range of different South African families, as well as to illustrate how particular constructions either promote or hinder the development of gender-equitable relations. This focus was informed by a large body of literature that suggests that particular problematic constructions of gender are linked to practices of violence and sexual risk. We are not arguing that all instances in which gender is constructed in problematic ways result in violence or sexual risk (and our study did not investigate the direct relationship between particular constructions of gender and these health outcomes). However, we believe, given the strong trend in the literature on gender and violence and sexual risk, it is necessary to develop a better understanding of how these constructions of gender operate, particular for these health outcomes.

The results of the study reveal that families exist as gendered spaces in which notions of gender are constructed and enacted in particular ways. Particularly striking are the ways in which problematic constructions of gender were both (re)produced and challenged simultaneously. This complex and contradictory process has important implications for how gender equality can be promoted within the context of families. Despite some families’ claims that they were ‘egalitarian/feminist’, the data revealed that problematic constructions of gender were drawn on within all 18 families. Hence, we contended that a way of approaching and categorising families is on a spectrum, from more patriarchal/less egalitarian to less patriarchal/more egalitarian. Although some families appear to be more successful at disrupting problematic constructions of gender, it is important to recognise the continued power of patriarchy in shaping both notions and practices of gender within South African families. In view of the enormous costs that are incurred as a result of gender inequality, not only in South Africa but also in a global context, it is important to more closely examine the mechanism through which families are able to successfully challenge gender inequality as well as the intersecting forces that resist gender transformation within families. Such an examination is likely to provide us with important clues as to how we can more effectively begin to dismantle hegemonic discourses that scaffold social practices of gender inequity in South Africa, as well as in other contexts.

Overall, the results suggest that there is a relationship between the ways in which parents and children construct gender, as illustrated by the examples of Elijah and Charmaine and Daniel and Nomhle. This points to the fact that the ways in which parents construct gender have an effect on their children's notions of gender and gender equality. This means that both gender inequality and gender equality are being (re)produced within families. However, there were also examples within some families where parents’ and children's constructions of gender contradicted each other. This was particularly true of children over the age of 13. While a more comprehensive exploration of the nature of these contradictions is beyond the scope of this paper, their occurrence in a number of families is noteworthy. These contradictions suggests that although the family represents an important site in which children are exposed to notions of gender, other spaces (for example peer groups, school, and social media) may also shape children's constructions of gender in meaningful ways. It is possible that this is particularly true for older children, as they are likely to spend more time and attribute more significance to these non-familial spaces. Therefore, there is a need to further investigate the ways in which these other spaces shape children's constructions of gender.

Equally importantly, the results of the study highlight how both the (re)production and challenging of problematic discourses of gender inequality are shaped in complex ways by various intersecting categories of identity (including gender, age, race, class, culture, and education). For example, we have argued that while in some instances poverty appears to produce problematic constructions of gender, for example in the cases of Caleb, Elijah and Charmaine, in other cases it appears to produce egalitarian constructions of gender, for example in the case of Pamela. These findings suggest that gender equality is possible in a variety of different contexts, a finding that has important implications for the promotion of gender equality in South Africa and possibly beyond.

## Footnotes

1 While the term *(re)produced* has a number of divergent meanings, within the context of the paper it is used to refer to the maintenance of inequitable gender relations. More specifically, we use the term to refer to the way in which ‘widely shared gender stereotypes act as a “common knowledge” cultural frame that people use to begin the process of relating to one another and coordinating their interaction’ (6, p. 2).

2 The case of Anene Booysen (7) received widespread media attention in South Africa, arguably due to the extremely violent nature of this attack. Booysen was raped, disemboweled, and left for dead at a building site in a rural town in the Western Cape. She died later the same day due to her injuries.

3 Jade Panayiotou, a primary school teacher, disappeared outside her middle-class Port Elizabeth home. She was later found murdered. This case received widespread coverage in the South African media.

4 The widespread media attention and outrage that emerged in response to the cases of Reeva Steenkamp and Jade Panayiotou has highlighted the ways in which certain instances of violence are seen as being more ‘horrifying’ and ‘unnatural’ than others.

5 A few children under the age of seven gave very short interviews. This was likely due to their developmental phase, which meant that they were unable to elaborate or explain clearly some of their responses. This may have also been exacerbated for children whose first language was not English. However, although some interviews were very short they nonetheless provided interesting data.

6 Pseudonyms have been used to protect the identities of all participants.

## Paper context

Research has established a strong link between the epidemics of violence and HIV and gender inequality. However, there is a lack of research exploring how inequality is (re)produced or challenged within everyday spaces such as the family. Our findings suggest that families operate as key sites in which problematic constructions of gender are both perpetuated and disrupted. In future, research should advance an understanding of how gender equality can be meaningfully promoted at home.
